# Supplementary figures and images for: The impact of intermittent preventive treatment in school aged children with dihydroartemisinin piperaquine and artesunate amodiaquine on IgG response against six blood stage Plasmodium falciparum antigens
Source: PLoS One. 2025 Jan 30;20(1):e0316482. doi: 10.1371/journal.pone.0316482 (PMC11781616; doi:10.1371/journal.pone.0316482)

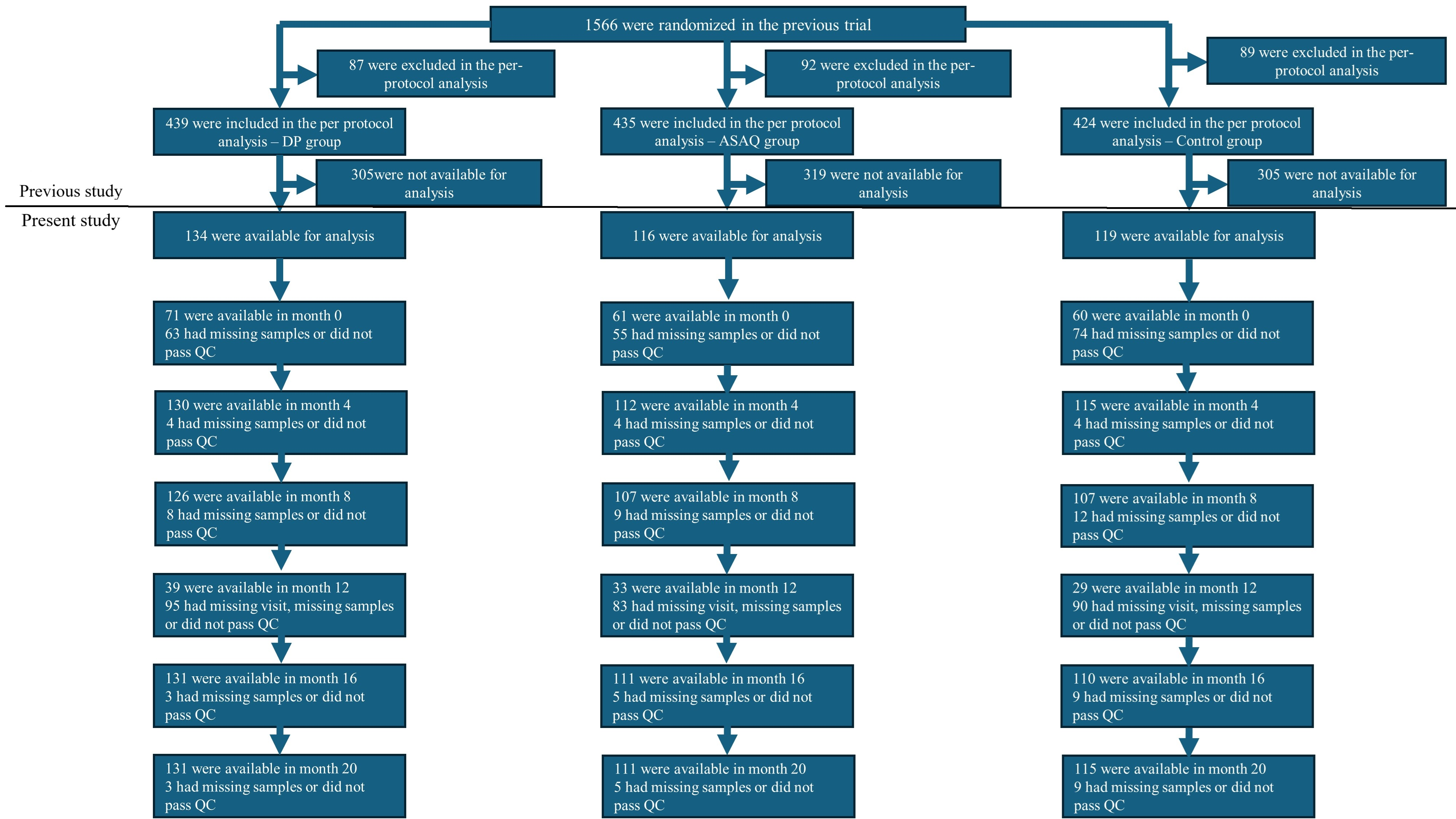

Supplement: S1 Fig — From the previous study by Makenga et al. [10], 1566 schoolchildren were enrolled in randomized, controlled, open-label trial. The present study analysed samples from 369 schoolchildren, 119 participants were in the control group, 134 in the DP group and 116 in the ASAQ group. (TIF) [file pone.0316482.s001.tif]

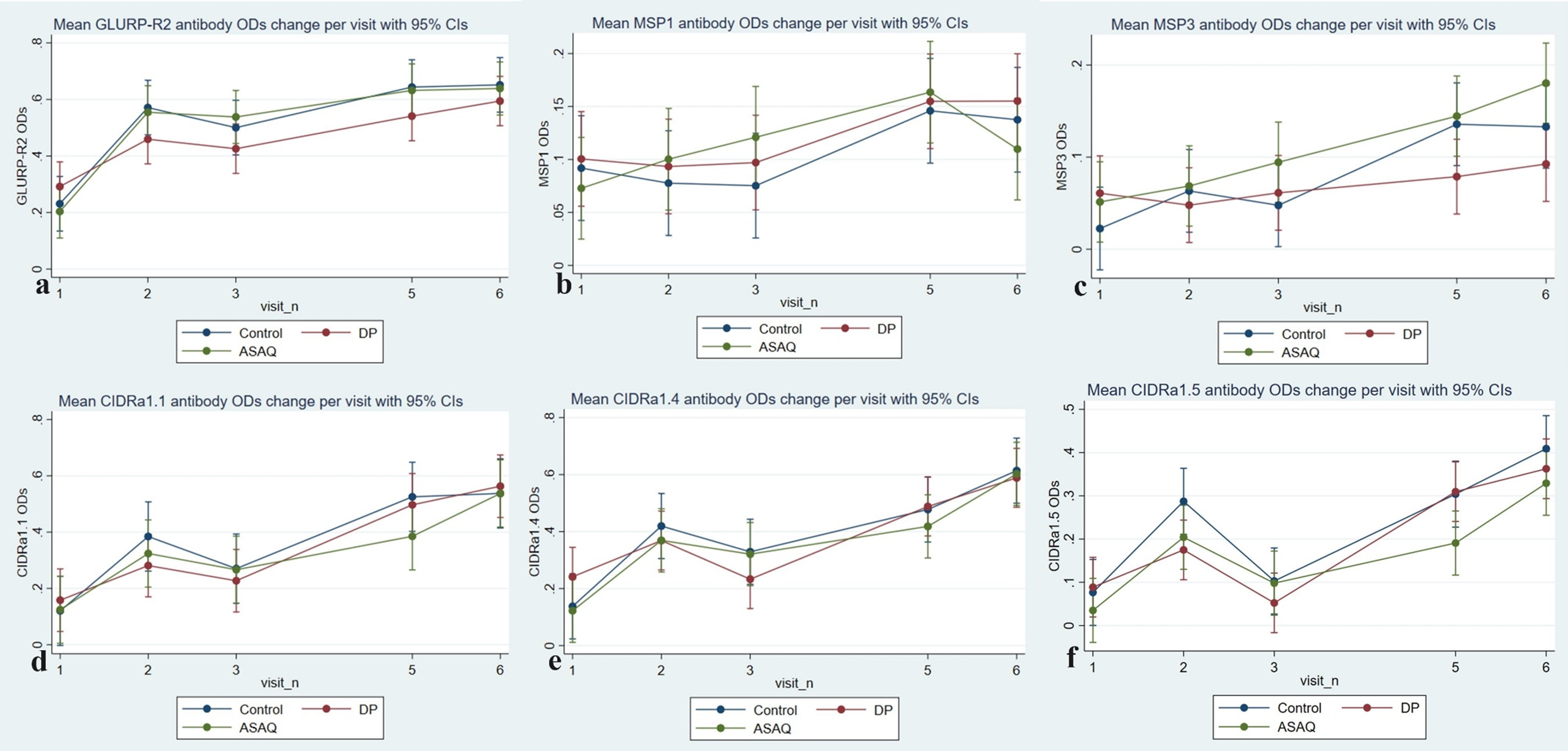

Supplement: S2 Fig — Figures showing participants who attended all visits, and visit 4 has been excluded in the analysis: (a) antibodies trend against the glutamate rich protein-region 2 (GLURP-R2), (b) antibodies trend against the merozoite surface protein 1 (MSP1), (c) antibodies trend against the merozoite surface protein 3 (MSP3), (d, e, f) antibodies trend against three cysteine-rich interdomain region (CIDR) domains (CIDRa1.1, CIDRa1.4 and CIDRa1.5) of P. falciparum erythrocyte membrane protein 1 (PfEMP1). (TIF) [file pone.0316482.s002.TIF]

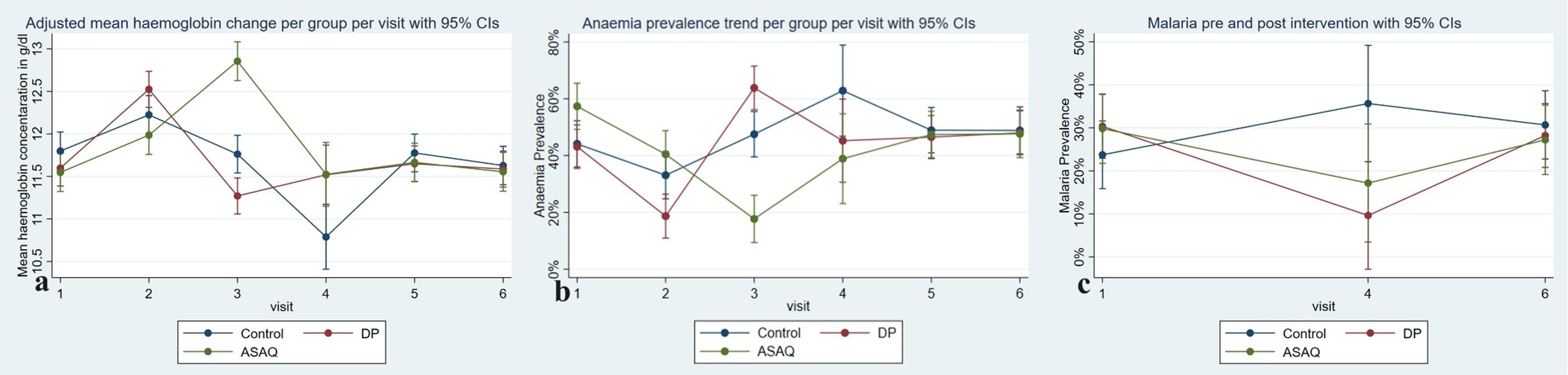

Supplement: S3 Fig — The impact of intermittent preventive treatment on malaria in school-aged children (IPTsc) on (a) change of mean haemoglobin, (b) anaemia prevalence and (c) malaria prevalence from baseline, at each visit per study group (n = 369). (TIF) [file pone.0316482.s003.TIF]
