# Supplementary material for: The impact of intermittent preventive treatment in school aged children with dihydroartemisinin piperaquine and artesunate amodiaquine on IgG response against six blood stage Plasmodium falciparum antigens
Source: PLoS One. 2025 Jan 30;20(1):e0316482. doi: 10.1371/journal.pone.0316482 (PMC11781616; doi:10.1371/journal.pone.0316482)
Supplement: S1 Table — (DOCX) [file pone.0316482.s004.docx]

# **Supporting information**

**S1 Table**. Linear mixed effect analysis on the impact of IPTsc intervention on antibody response against six *P. falciparum* antigens in schoolchildren aged between 5 – 15 years, n=369.

| Study visit (Month) | DP vs control groups | | ASAQ vs  control groups | |
| --- | --- | --- | --- | --- |
|  | Difference in mean change in Optical densities from baseline, (95% CI) | p value | Difference in mean change in optical densities from baseline, (95% CI) | p value |
| **GLURP R2** | | | | |
| 1 (0) Baseline | -- | -- | -- | -- |
| 2 (4) | -0.13 (-0.22 – -0.04) | **0.004** | -0.01 (-0.10 – 0.08) | 0.88 |
| 3 (8) | -0.10 (-0.18 – -0.01) | **0.031** | 0.08 (-0.02 – 0.17) | 0.10 |
| 4 (12) | -0.11 (-0.24 – 0.02) | 0.09 | -0.07 (-0.20 – 0.06) | 0.31 |
| 5 (16) | -0.09 (-0.18 – -0.01) | **0.035** | 0.06 (-0.03 – 0.15) | 0.19 |
| 6 (20) | -0.12 (-0.21 – -0.04) | **0.006** | 0.03 (-0.06 – 0.12) | 0.53 |
|  |  |  |  |  |
| **MSP3** | | | | |
| 1 (0) Baseline | -- | -- | -- | -- |
| 2 (4) | -0.04 (-0.09 – 0.02) | 0.18 | -0.01 (-0.06 – 0.05) | 0.85 |
| 3 (8) | -0.01 (-0.07 – 0.05) | 0.72 | 0.04 (-0.02 – 0.10) | 0.22 |
| 4 (12) | -0.04 (-0.12 – 0.04) | 0.36 | 0.01 (-0.08 – 0.09) | 0.89 |
| 5 (16) | -0.04 (-0.10 – 0.01) | 0.12 | -0.00 (-0.06 – 0.06) | 0.92 |
| 6 (20) | -0.06 (-0.12 – -0.01) | **0.039** | 0.02 (-0.04 – 0.08) | 0.47 |
|  |  |  |  |  |
| **MSP1** | | | | |
| 1 (0) Baseline | -- | -- | -- | -- |
| 2 (4) | 0.02 (-0.03 – 0.07) | 0.47 | 0.03 (-0.02 – 0.08) | 0.17 |
| 3 (8) | 0.00 (-0.04 – 0.05) | 0.87 | 0.04 (-0.01 – 0.09) | 0.12 |
| 4 (12) | 0.04 (-0.03 – 0.11) | 0.21 | 0.04 (-0.03 – 0.11) | 0.28 |
| 5 (16) | 0.02 (-0.03 – 0.07) | 0.51 | 0.04 (-0.01 – 0.09) | 0.12 |
| 6 (20) | -0.01 (-0.05 – 0.04) | 0.81 | -0.01 (-0.06 – 0.04) | 0.76 |
|  |  |  |  |  |
| **CIDRa1.1** | | | | |
| 1 (0) Baseline | -- | -- | -- | -- |
| 2 (4) | -0.09 (-0.22 – 0.04) | 0.16 | -0.03 (-0.17 – 0.10) | 0.61 |
| 3 (8) | -0.05 (-0.17 – 0.08) | 0.49 | -0.02 (-0.16 – 0.11) | 0.73 |
| 4 (12) | -0.13 (-0.31 – 0.05) | 0.17 | -0.16 (-0.35 – 0.03) | 0.10 |
| 5 (16) | -0.06 (-0.18 – 0.07) | 0.40 | -0.07 (-0.20 – 0.06) | 0.29 |
| 6 (20) | -0.00 (-0.13 – 0.12) | 0.97 | 0.01 (-0.12 – 0.15) | 0.82 |
|  |  |  |  |  |
| **CIDRa1.4** | | | | |
| 1 (0) Baseline | -- | -- | -- | -- |
| 2 (4) | -0.11 (-0.23 – 0.01) | 0.07 | 0.00 (-0.12 – 0.13) | 0.98 |
| 3 (8) | -0.11 (-0.23 – 0.02) | 0.09 | 0.02 (-0.11 – 0.14) | 0.80 |
| 4 (12) | -0.11 (-0.28 – 0.07) | 0.23 | -0.06 (-0.24 – 0.12) | 0.51 |
| 5 (16) | -0.08 (-0.20 – 0.04) | 0.21 | -0.01 (-0.14 – 0.11) | 0.83 |
| 6 (20) | -0.20 (-0.32 – -0.08) | **0.001** | -0.04 (-0.17 – 0.09) | 0.53 |
|  |  |  |  |  |
| **CIDRa1.5** | | | | |
| 1 (0) Baseline | -- | -- | -- | -- |
| 2 (4) | -0.07 (-0.17 – 0.04) | 0.20 | -0.00 (-0.11 – 0.11) | 1.00 |
| 3 (8) | -0.04 (-0.15 – 0.06) | 0.42 | 0.03 (-0.08 – 0.14) | 0.61 |
| 4 (12) | -0.02 (-0.16 – 0.13) | 0.82 | -0.00 (-0.15 – 0.15) | 0.97 |
| 5 (16) | -0.01 (-0.11 – 0.10) | 0.91 | -0.01 (-0.11 – 0.10) | 0.91 |
| 6 (20) | -0.10 (-0.20 – 0.00) | **0.050** | -0.06 (-0.16 – 0.05) | 0.31 |
