# Supplementary material for: The impact of intermittent preventive treatment in school aged children with dihydroartemisinin piperaquine and artesunate amodiaquine on IgG response against six blood stage Plasmodium falciparum antigens
Source: PLoS One. 2025 Jan 30;20(1):e0316482. doi: 10.1371/journal.pone.0316482 (PMC11781616; doi:10.1371/journal.pone.0316482)
Supplement: S2 Table — Analysis based on participants who attended all visits, and visit 4 has been excluded in the analysis, n = 161. (DOCX) [file pone.0316482.s005.docx]

# **Supporting information**

**S2 Table**. Linear mixed effect analysis on the impact of IPTsc intervention on antibody response against six *P. falciparum* antigens in schoolchildren aged between 5 – 15 years. Analysis based on participants who attended all visits, and visit 4 has been excluded in the analysis, n = 161.

| Intervention period | DP vs control groups | | ASAQ vs  control groups | |
| --- | --- | --- | --- | --- |
|  | Difference in mean change in Optical densities from baseline, (95% CI) | p value | Difference in mean change in optical densities from baseline, (95% CI) | p value |
| **GLURP R2** | | | | |
| 1 (0) Baseline | -- | -- | -- | -- |
| 2 (4) | -0.17 (-0.28 – -0.07) | **0.001** | 0.01 ( -0.10 – 0.12) | 0.84 |
| 3 (8) | -0.14 ( -0.24 – -0.03) | **0.011** | 0.07 ( -0.04 – 0.17) | 0.24 |
| 4 (12) | NA | NA | NA | NA |
| 5 (16) | -0.16 ( -0.27 – -0.06) | **0.002** | 0.02 ( -0.09 – 0.12) | 0.78 |
| 6 (20) | -0.12 ( -0.22 – -0.01) | **0.027** | 0.01 ( -0.09 – 0.12) | 0.79 |
|  |  |  |  |  |
| **MSP3** | | | | |
| 1 (0) Baseline | -- | -- | -- | -- |
| 2 (4) | -0.05 (-0.13 – 0.02) | 0.15 | -0.02 (-0.10 – 0.05) | 0.54 |
| 3 (8) | -0.02 (-0.10 – 0.05) | 0.50 | 0.02 (-0.06 – 0.09) | 0.64 |
| 4 (12) | NA | NA | NA | NA |
| 5 (16) | -0.10 (-0.17 – -0.02) | **0.010** | -0.02 (-0.09 – 0.05) | 0.60 |
| 6 (20) | -0.08 (-0.15 – -0.01) | **0.033** | 0.02 (-0.06 – 0.09) | 0.63 |
|  |  |  |  |  |
| **MSP1** | | | | |
| 1 (0) Baseline | -- | -- | -- | -- |
| 2 (4) | 0.01 (-0.05 – 0.06) | 0.81 | 0.04 (-0.02 – 0.10) | 0.17 |
| 3 (8) | 0.01 (-0.04 – 0.07) | 0.65 | 0.06 (0.01 – 0.12) | 0.03 |
| 4 (12) | NA | NA | NA | NA |
| 5 (16) | 0.00 (-0.06 – 0.06) | 0.99 | 0.04 (-0.02 – 0.10) | 0.23 |
| 6 (20) | 0.01 (-0.05 – 0.07) | 0.76 | -0.01 (-0.07 – 0.05) | 0.78 |
|  |  |  |  |  |
| **CIDRa1.1** | | | | |
| 1 (0) Baseline | -- | -- | -- | -- |
| 2 (4) | -0.14 (-0.30 – 0.02) | 0.08 | -0.06 (-0.23 – 0.10) | 0.44 |
| 3 (8) | -0.08 (-0.24 – 0.08) | 0.32 | -0.01 (-0.17 – 0.16) | 0.93 |
| 4 (12) | NA | NA | NA | NA |
| 5 (16) | -0.07 (-0.22 – 0.09) | 0.41 | -0.14 (-0.31 – 0.02) | 0.09 |
| 6 (20) | -0.01 (-0.17 – 0.15) | 0.88 | 0.00 (-0.17 – 0.16) | 0.96 |
|  |  |  |  |  |
| **CIDRa1.4** | | | | |
| 1 (0) Baseline | -- | -- | -- | -- |
| 2 (4) | -0.15 (-0.31 – 0.00) | 0.05 | -0.03 (-0.20 – 0.13) | 0.67 |
| 3 (8) | -0.20 (-0.36 – -0.04) | 0.01 | 0.01 (-0.15 – 0.17) | 0.93 |
| 4 (12) | NA | NA | NA | NA |
| 5 (16) | -0.09 (-0.25 – 0.06) | 0.24 | -0.04 (-0.21 – 0.12) | 0.59 |
| 6 (20) | -0.13 (-0.29 – 0.03) | 0.11 | 0.00 (-0.16 – 0.17) | 0.96 |
|  |  |  |  |  |
| **CIDRa1.5** | | | | |
| 1 (0) Baseline | -- | -- | -- | -- |
| 2 (4) | -0.12 (-0.26 – 0.01) | 0.06 | -0.04 (-0.18 – 0.09) | 0.55 |
| 3 (8) | -0.06 (-0.19 – 0.07) | 0.35 | 0.04 (-0.10 – 0.17) | 0.60 |
| 4 (12) | NA | NA | NA | NA |
| 5 (16) | -0.01 (-0.14 – 0.12) | 0.92 | -0.07 (-0.21 – 0.06) | 0.30 |
| 6 (20) | -0.06 (-0.19 – 0.07) | 0.38 | -0.04 (-0.17 – 0.10) | 0.58 |
