# Supplementary material for: The impact of intermittent preventive treatment in school aged children with dihydroartemisinin piperaquine and artesunate amodiaquine on IgG response against six blood stage Plasmodium falciparum antigens
Source: PLoS One. 2025 Jan 30;20(1):e0316482. doi: 10.1371/journal.pone.0316482 (PMC11781616; doi:10.1371/journal.pone.0316482)
Supplement: Attachment — Submitted filename: PONE-D-24-14848_reviewer.pdf [file pone.0316482.s009.pdf]

PLOS ONE

The impact of Intermittent Preventive Treatment among School aged Children with Dihydroartemisinin Piperaquine and Artesunate Amodiaquine on IgG response against six blood stage Plasmodium falciparum antigens

--Manuscript Draft--

**Manuscript Number:**	PONE-D-24-14848
**Article Type:**	Research Article
**Full Title:**	The impact of Intermittent Preventive Treatment among School aged Children with Dihydroartemisinin Piperaquine and Artesunate Amodiaquine on IgG response against six blood stage Plasmodium falciparum antigens
**Short Title:**	The impact of Intermittent Preventive Treatment with DP and ASAQ on IgG response
**Corresponding Author:**	Eric Lyimo, MSc<br>NIMR: National Institute for Medical Research<br>Mwanza, TANZANIA, UNITED REPUBLIC OF
**Keywords:**	Intermittent preventive treatment; schoolchildren; dihydroartemisinin-piperaquine; artesunate-amodiaquine; Malaria
**Abstract:**	Several interventional strategies have been implemented in malaria endemic areas where the burden is high, that include among others, intermittent preventive treatment (IPT), a tactic that blocks transmission and can reduce disease morbidity. However, the implementation IPT strategies raises a genuine concern, intervening the development of naturally acquired immunity to malaria which requires continuous contact with parasite antigens. This study investigated whether dihydroartemisinin-piperaquine (DP) or artesunate-amodiaquine (ASAQ) IPT in schoolchildren (IPTsc) impairs IgG reactivity to six malaria antigens. An IPTsc trial in north-eastern Tanzania administered three doses of DP or ASAQ four months apart, the schoolchildren were followed up. This study compared IgG reactivity against GLURP-R2, MSP1, MSP3, and CIDR domains (CIDRα1.1, CIDRα1.4, and CIDRα1.5) of Plasmodium falciparum erythrocyte membrane protein 1 (PfEMP-1) in intervention and control groups using enzyme linked immunosorbent assay (ELISA) technique. During the study, 369 schoolchildren were available for analysis, 119, 134 and 116 participants in the control, DP and ASAQ groups, respectively. Breadth of malaria antigen recognition increased significantly during and after the intervention phases and did not differ between the study groups (Trend test: DP, z-score = 5.92, $p < 0.001$, ASAQ, z-score = 6.64, $p < 0.001$ and control, z-score = 5.85, $p < 0.001$). There were no differences between the control and ASAQ group in the recognition of any of the tested antigens at all visits. In the DP group, however, during the intervention period IPTsc did not impair antibody against MSP1, MSP3, CIDRα1.1, CIDRα1.4 and CIDRα1.5, but it did impair against GLURP-R2. The current study has shown that effective IPTsc with DP or ASAQ does not interfere with the development of antibodies against malaria antigens of the blood stages, suggesting that the advancement of naturally acquired immunity to malaria is not impeded by IPTsc interventions.
**Order of Authors:**	Eric Lyimo, MSc
	Geofrey Makenga, PhD
	Louise Turner, PhD
	Thomas Lavstsen, PhD
	John P. A. Lusingu, PhD
	Jean-Pierre Van geertruyden, PhD
	Daniel T. R. Minja, PhD
	Christian W. Wang, PhD
	Vito Baraka, PhD
**Additional Information:**	

Powered by Editorial Manager® and ProduXion Manager® from Aries Systems Corporation

Question	Response
**Financial Disclosure**<br><br>Enter a financial disclosure statement that describes the sources of funding for the work included in this submission. Review the submission guidelines for detailed requirements. View published research articles from *PLOS ONE* for specific examples.<br><br>This statement is required for submission and **will appear in the published article** if the submission is accepted. Please make sure it is accurate.<br><br>**Funded studies**<br>Enter a statement with the following details:<br>• Initials of the authors who received each award<br>• Grant numbers awarded to each author<br>• The full name of each funder<br>• URL of each funder website<br>• Did the sponsors or funders play any role in the study design, data collection and analysis, decision to publish, or preparation of the manuscript?<br><br>Did you receive funding for this work?	Yes
Please add funding details.<br>as follow-up to "**Financial Disclosure**<br><br>Enter a financial disclosure statement that describes the sources of funding for the work included in this submission. Review the submission guidelines for detailed requirements. View published research articles from *PLOS ONE* for specific examples.<br><br>This statement is required for submission and **will appear in the published article** if the submission is accepted. Please make sure it is accurate.	The Flemish Interuniversity Council (VLIRUOS), Belgium (TEAM initiative, grant number TZ2017TEA451A102 awarded to GM), funded the main study, the clinical trial. This study was funded by the Malaria Research and Capacity Building for field trials in Tanzania (MaReCa) project as part of the EDCTP2 programme supported by the European Union (Grant number TMA2015SF–998-MaReCa awarded to JPAL).

**Funded studies**<br>Enter a statement with the following details:<br>• Initials of the authors who received each award<br>• Grant numbers awarded to each author<br>• The full name of each funder<br>• URL of each funder website<br>• Did the sponsors or funders play any role in the study design, data collection and analysis, decision to publish, or preparation of the manuscript?<br><br>Did you receive funding for this work?"	
Please select the country of your main research funder (please select carefully as in some cases this is used in fee calculation).<br>as follow-up to "**Financial Disclosure**<br><br>Enter a financial disclosure statement that describes the sources of funding for the work included in this submission. Review the submission guidelines for detailed requirements. View published research articles from *PLOS ONE* for specific examples.<br><br>This statement is required for submission and **will appear in the published article** if the submission is accepted. Please make sure it is accurate.<br><br>**Funded studies**<br>Enter a statement with the following details:<br>• Initials of the authors who received each award<br>• Grant numbers awarded to each author<br>• The full name of each funder<br>• URL of each funder website<br>• Did the sponsors or funders play any role in the study design, data collection and analysis, decision to publish, or preparation of the manuscript?<br><br>Did you receive funding for this work?"	TANZANIA, UNITED REPUBLIC OF - TZ
**Competing Interests**	The authors have declared that no competing interests exist.

Use the instructions below to enter a competing interest statement for this submission. On behalf of all authors, disclose any competing interests that could be perceived to bias this work—acknowledging all financial support and any other relevant financial or non-financial competing interests.<br><br>This statement is required for submission and **will appear in the published article** if the submission is accepted. Please make sure it is accurate and that any funding sources listed in your Funding Information later in the submission form are also declared in your Financial Disclosure statement.<br><br>View published research articles from *PLOS ONE* for specific examples.<br><br>**NO authors have competing interests**<br><br>Enter: *The authors have declared that no competing interests exist.*<br><br>**Authors with competing interests**<br><br>Enter competing interest details beginning with this statement:<br><br>*I have read the journal's policy and the authors of this manuscript have the following competing interests: [insert competing interests here]*<br><br>* typeset	
**Ethics Statement**<br><br>Enter an ethics statement for this submission. This statement is required if the study involved:<br><br>• Human participants<br>• Human specimens or tissue<br>• Vertebrate animals or cephalopods<br>• Vertebrate embryos or tissues	The study received ethical approval from the Medical Research Coordinating Committee of Tanzania with reference number, NIMR/HQ/R.8a/Vol.IX/2818, NIMR/HQ/R.8c/Vol.I/668 for amendment, and NIMR/HQ/ R.8c/Vol.I/1276 for renewal. Also, the study received authorization from the local government authorities and local school committees. Parents or guardians provided written informed consent for their children to participate, while children aged 11 or older were requested to assent. The study also received regulatory approval from the Tanzania Medicines and Medical Devices Authority with reference number TFDA0017/CTR/0018/07.

• Field research<br><br>Write "N/A" if the submission does not require an ethics statement.<br><br>General guidance is provided below. Consult the submission guidelines for detailed instructions. **Make sure that all information entered here is included in the Methods section of the manuscript.**	

**Format for specific study types**<br><br>**Human Subject Research (involving human participants and/or tissue)**<br>• Give the name of the institutional review board or ethics committee that approved the study<br>• Include the approval number and/or a statement indicating approval of this research<br>• Indicate the form of consent obtained (written/oral) or the reason that consent was not obtained (e.g. the data were analyzed anonymously)<br><br>**Animal Research (involving vertebrate animals, embryos or tissues)**<br>• Provide the name of the Institutional Animal Care and Use Committee (IACUC) or other relevant ethics board that reviewed the study protocol, and indicate whether they approved this research or granted a formal waiver of ethical approval<br>• Include an approval number if one was obtained<br>• If the study involved *non-human primates*, add *additional details* about animal welfare and steps taken to ameliorate suffering<br>• If anesthesia, euthanasia, or any kind of animal sacrifice is part of the study, include briefly which substances and/or methods were applied<br><br>**Field Research**<br><br>Include the following details if this study involves the collection of plant, animal, or other materials from a natural setting:<br>• Field permit number<br>• Name of the institution or relevant body that granted permission	
**Data Availability**<br><br>Authors are required to make all data underlying the findings described fully available, without restriction, and from the time of publication. PLOS allows rare exceptions to address legal and ethical concerns. See the PLOS Data Policy and FAQ for detailed information.	No - some restrictions will apply

A Data Availability Statement describing where the data can be found is required at submission. Your answers to this question constitute the Data Availability Statement and **will be published in the article**, if accepted.<br><br>**Important:** Stating 'data available on request from the author' is not sufficient. If your data are only available upon request, select 'No' for the first question and explain your exceptional situation in the text box.<br><br>Do the authors confirm that all data underlying the findings described in their manuscript are fully available without restriction?	
**Describe where the data may be found in full sentences. If you are copying our sample text, replace any instances of XXX with the appropriate details.**<br><br>• If the data are **held or will be held in a public repository**, include URLs, accession numbers or DOIs. If this information will only be available after acceptance, indicate this by ticking the box below. For example: *All XXX files are available from the XXX database (accession number(s) XXX, XXX.)*.<br>• If the data are all contained **within the manuscript and/or Supporting Information files**, enter the following: *All relevant data are within the manuscript and its Supporting Information files.*<br>• If neither of these applies but you are able to provide **details of access elsewhere**, with or without limitations, please do so. For example:<br><br>*Data cannot be shared publicly because of [XXX]. Data are available from the XXX Institutional Data Access / Ethics Committee (contact via XXX) for researchers who meet the criteria for access to confidential data.*<br><br>*The data underlying the results presented in the study are available from (include the name of the third party*	The full datasets used and analysed in this study are available from the corresponding author (EL) on reasonable request and following the Ethics Committee and institutional data transfer policy.

and contact information or URL).<br>• This text is appropriate if the data are owned by a third party and authors do not have permission to share the data.<br><br>* typeset	
Additional data availability information:	

Powered by Editorial Manager® and ProduXion Manager® from Aries Systems Corporation

Manuscript
Click here to access/download;Manuscript;Manuscript The impact of IPTsc with DP and ASAQ_Final.docx

The impact of Intermittent Preventive Treatment among School aged Children with Dihydroartemisinin Piperaquine and Artesunate Amodiaquine on IgG response against six blood stage *Plasmodium falciparum* antigens

Eric Lyimo[1,2,3 *], Geofrey Makenga[1,4], Louise Turner[3,5], Thomas Lavstsen[3,5], John P. A. Lusingu[1], Jean-Pierre Van geertruyden[4], Daniel T. R. Minja[1], Christian W. Wang[3,5,¶], Vito Baraka[1,¶]

[1]National Institute for Medical Research, Tanga Research Centre, Tanzania

[2]National Institute for Medical Research, Mwanza Research Centre, Tanzania

[3]Department of Immunology and Microbiology, Centre for translational Medicine and Parasitology, University of Copenhagen, Copenhagen, Denmark.

[4]Global Health Institute, University of Antwerp, Antwerp, Belgium

[5]Department of Infectious Diseases, Copenhagen University Hospital, Copenhagen, Denmark.

[*]Corresponding author

E-mail: ericlyimo@gmail.com (EL)

[¶]These authors contributed equally to this work.

Abstract

Several interventional strategies have been implemented in malaria endemic areas where the burden is high, that include among others, intermittent preventive treatment (IPT), a tactic that blocks transmission and can reduce disease morbidity. However, the implementation IPT strategies raises a genuine concern, intervening the development of naturally acquired immunity to malaria which requires continuous contact with parasite antigens. This study investigated whether dihydroartemisinin-piperaquine (DP) or artesunate-amodiaquine (ASAQ) IPT in schoolchildren (IPTsc) impairs IgG reactivity to six malaria antigens. An IPTsc trial in north-eastern Tanzania administered three doses of DP or ASAQ four months apart, the schoolchildren were followed up. This study compared IgG reactivity against GLURP-R2, MSP1, MSP3, and CIDR domains (CIDRα1.1, CIDRα1.4, and CIDRα1.5) of *Plasmodium falciparum* erythrocyte membrane protein 1 (PfEMP-1) in intervention and control groups using enzyme linked immunosorbent assay (ELISA) technique. During the study, 369 schoolchildren were available for analysis, 119, 134 and 116 participants in the control, DP and ASAQ groups, respectively. Breadth of malaria antigen recognition increased significantly during and after the intervention phases and did not differ between the study groups (Trend test: DP, z-score = 5.92, $p < 0.001$, ASAQ, z-score = 6.64, $p < 0.001$ and control, z-score = 5.85, $p < 0.001$). There were no differences between the control and ASAQ group in the recognition of any of the tested antigens at all visits. In the DP group, however, during the intervention period IPTsc did not impair antibody against MSP1, MSP3, CIDRα1.1, CIDRα1.4 and CIDRα1.5, but it did impair against GLURP-R2. The current study has shown that effective IPTsc with DP or ASAQ does not interfere with the development of antibodies against malaria antigens of the blood stages, suggesting that the advancement of naturally acquired immunity to malaria is not impeded by IPTsc interventions.

Introduction

Malaria is still a major global health problem, with an estimated 249 million cases and 608,000 deaths in 2022, and Tanzania is among the four countries that accounted for over half of all global deaths [1]. The fight against malaria mortality and morbidity has been moderately achieved with several strategies, such as the widespread use of insecticide-treated mosquito nets (ITNs) and indoor residual spraying (IRS), aiming to control the malaria vector. Additional strategies include intermittent preventive treatment (IPT), seasonal malaria chemoprevention (SMC) for transmission intervention, malaria rapid diagnostic tests (mRDTs) used at the community level and widespread use of artemisinin-based combination therapies (ACTs) for the treatment of *Plasmodium falciparum* infection. In areas with high malaria transmission, the WHO recommends IPT for high-risk groups, infants and pregnant women, and SMC for children, which is also referred to as intermittent preventive treatment in children (IPTsc) [2, 3]. However, following the application of efficient measures to control malaria in high-risk groups, including children under five, the malaria burden has broadened to include school age children, above 5 years of age [4, 5]. The schoolchildren have recently captured the attention of researchers after being identified as a group that have the highest *P. falciparum* infection rate but with low mortality and morbidity making them a prominent reservoir for transmission [4, 6, 7]. Recent reviews have recommended intermittent preventive treatment in schoolchildren (IPTsc) as the optimal option in terms of acceptability, feasibility that may reduce malaria burden in schoolchildren residing in high transmission areas [6, 8]. Clinical trials have shown that IPTsc with sulphadoxine-pyrimethamine (SP) [9] or ACTs [10] are safe and effective when administered every four months for one year, and the efficacy could last one year after the last dose [10].

The use of IPT though, raises a vital concern that it may hamper the development of naturally acquired immunity to malaria. Immunity to disease develops with continued exposure to

malaria antigens and IPT may reduce this exposure [11] and hence when chemoprophylaxis is stopped, the risk of malaria increases [12]. Previous studies have investigated the impact of IPT on antibody development: Intermittent preventive treatment in pregnancy (IPTp) and in infants (IPTi) with sulphadoxine-pyrimethamine (SP) [13, 14], IPTc with artesunate plus SP [15], and found that IPT reduces antibody development against *P. falciparum*. Yet again, other studies have found no effect on the antibody development after IPTi with SP [14, 16].

In Tanzania, SP has long been barred as a first line treatment option because of circulating SP resistant parasites [17, 18]. Therefore, the use of effective artemisinin and artemisinin-derivatives for IPT in combination with other drugs are appealing and in a study by Makenga et al., [10], it was found that IPTsc with dihydroartemisinin-piperaquine (DP) or artesunate-amodiaquine (ASAQ) delivered in three doses every four months is effective in subsiding *P. falciparum* parasitaemia and reducing clinical malaria and related morbidities 12 months after the first dose.

In this present study, we investigated the IgG reactivity to six different antigens of *P. falciparum* among schoolchildren in the IPTsc study by Makenga et al [10] during and after intervention. Three cysteine-rich interdomain region (CIDR) domains (CIDRα1.1, CIDRα1.4 and CIDRα1.5) of *P. falciparum* erythrocyte membrane protein 1 (PfEMP1), expressed on the surface enabling the sequestration of infected erythrocytes to the endothelium of capillaries and venules and associated with severe malaria [19, 20], and three proteins associated with the parasite's merozoite stage: glutamate-rich protein region 2 (GLURP-R2) and merozoite surface protein 1 (MSP1) and 3 (MSP3). Antibodies against CIDRa1 domains have been shown to be associated with development of immunity against severe malaria and are acquired early in life [21, 22] however, lost if transmission is interrupted indicating that continued exposure is required to maintain the production of the antibodies [22]. Furthermore, antibodies against GLURP-R2, MSP1, MSP3 and other merozoite antigens

have been shown to control asymptomatic malaria infections [23]. Antibodies against GLURP have been associated with reduced risk of clinical malaria in Burkinabe and Ghanian children [24] and associated with the reduction in parasitaemia and febrile episodes of malaria in high and low malaria transmission areas [25, 26].

Materials and Methods

Study design, site, and participants

A previous study enrolled 1,566 schoolchildren in a randomised, controlled, open-label trial study conducted in Muheza District, Tanga, north-eastern Tanzania (G. Makenga et al., 2023), the trial was registered with ClinicalTrials.gov (NCT03640403). This study analysed samples from 369 schoolchildren who were seen in at in more than one visit and had samples that passed the quality control. In brief, the trial assessed the effectiveness and safety of dihydroartemisinin-piperaquine (DP) and artesunate-amodiaquine (ASAQ) as intermittent preventive treatment for malaria in school-aged children (IPTsc) in highly malaria-endemic areas. The study had six visits four months apart and baseline was between 26 March and 30 April 2019, and the last visit was completed on 31 December 2020. The study drugs were administered at baseline (month 0), the second (month 4), and the third visit (month 8). Participants were followed up, and no study drugs were given in the last three visits (months 12, 16, and 20). This study retrieved and accessed the data from the main trial in October 2022, and this study had no access to data that could identify individual participants. The main trial study objectives were to assess mean haemoglobin concentration change, clinical incidence of malaria, and parasitaemia prevalence from baseline at months 12 and 20 in the intervention groups against the control group.

The children were recruited from seven primary schools located in seven different villages in Muheza District, Tanga, north-eastern Tanzania. The study included schoolchildren between 5 and 15 years old with no signs of malaria or clinical features of anaemia. Each of the seven villages had a single primary school namely: Bwitini, Heinkele, Kwakibuyu, Mhamba, Mkulumilo, Pangamlima, and Songa Kibaoni. Children in class five or lower from each school were enrolled and randomly assigned to the three study arms. Children aged 11 or older provided informed assent, whereas informed consent from parents or guardians was obtained from children less than 11 years. The study area has two distinct periods of peak malaria transmission, the first occurs after the heavy rains season from March to May, while the second follows the moderate rains from December to January. The total study period from baseline to visit six was 20 months [10, 27], the first visit was conducted between March and April 2019, during which the study area experienced a long rainy season and before malaria transmission reached its highest rate. The study drugs were given at visit one (month 0) after enrolment, visit two (month 4) and at visit three (month 8). Visit two was conducted in August 2019 when malaria transmission was at its first highest annual peak (spanning between July and August) and visit three was conducted in January 2020 after a short rainy season when transmission was in its second annual peak (spanning between December and January) [28]. After the first year of three intervention visits, the schoolchildren were visited three times in the second year, visit four (month 12), visit five (month 16) and visit six (month 20) and no study drugs were administered, and during the fourth visit which was conducted in March 2020 and due to Severe Acute Respiratory Syndrome-2 (SARS-CoV-2) outbreak and WHO declaring the Coronavirus disease (COVID-19) pandemic, only two schools were visited. The Pangamlima and Songa Kibaoni schools were the only schools visited by the study team before the closure of public gatherings and activities.

Sample collection and processing

The schoolchildren provided finger prick blood samples at baseline, and in all other visits, dried blood spot (DBS) samples were made on Whatman 3 MM paper and haemoglobin concentration was measured with HemoCue® haemoglobin analyser (HemoCue AB, Angelholm, Sweden). The DBS samples were air dried overnight, stored in a Ziplock bag with desiccants, and then stored at -20°C until testing. At baseline, before treatment, thick and thin blood smears were made to determine parasitaemia. A total of 1,699 DBS samples from 369 schoolchildren were available for the current study.

GLURP-R2, MSP1, MSP3 and PfEMP1 IgG ELISA

We assessed the antibody recognition of six antigens of *P. falciparum* from the 1,699 available DBS samples. The sandwich enzyme-linked immunosorbent assay (ELISA) method was used to assess antibody recognition of GLURP-R2, MSP1, MSP3 and three PfEMP1 domains (CIDRa1.1, CIDRa1.4 and CIDRa1.5) as previously described [29, 30], with slight modifications. To elute IgG from DBS, a 6.35 mm hole puncher (Staples 10573-CC) was used to cut two DBS discs into a 96-deep-well plate which was then incubated with phosphate buffered saline (PBS) overnight at 4°C. The quality of elute was assessed as previously described [31], DBS discs that remained reddish-brown with pale PBS indicated incomplete elution and those samples were not included in the analysis. The DBS eluates were diluted 1:100 in PBS and then the ELISA assay was carried out in a 96-well microtiter plates (Nunc MaxiSorp). Initially, for every antigen the microtiter plates were coated with 5 µg/mL overnight at 4°C and blocked with 3% skimmed milk in PBS, after one hour the microtiter plates were washed three times and then 50 µL of samples were added to each well. Rabbit anti-human IgG HRP conjugated antibody (Dako, P0214) was added (1:3000) and incubated for 1 hour at room temperature to identify bound human IgG then the

microtiter plates were washed three times. Afterwards, 3,3',5,5' tetramethylbenzidine (TMB, T5525, Sigma-Aldrich) was added to detect bound human IgG. Optical densities (ODs) were measured using a Multiskan FC microtiter plate reader (Thermo Scientific) at 450 nm. DBS samples from naïve Danish blood donors were used as negative controls in the analysis to determine seropositivity, and values greater than three standard deviations from the mean of the naïve samples were used as the cut-off. Each participant's breadth of malaria antigen recognition was scored from 0 to 6 based on their IgG reactivity to the six antigens, as previously described in [32].

Statistical analysis

Data from the baseline survey and follow-up visits were managed as previously described [10], further data were analysed in STATA version 15.0, Microsoft Excel, and R 4.3.1 statistical software. Antibody responses to the six *P. falciparum* antigen were analysed as ELISA optical densities (ODs), first, the breadth of malaria antigen recognition was analysed as a binary response to the six antigens. Trend test across ordered groups was used to assess the trend in serorecognition across the study visits. Secondly, the antibody levels were analysed as continuous variables for each of the six targets, data were set as panel data in STATA and longitudinally analysed using a linear mixed model. Additionally, haemoglobin concentrations were also analysed using the linear mixed model. The model compared the changes within an individual subject and between study groups. The schoolchildren cohorts as observed in this study, usually have high rates of loss to follow-up but the linear mixed model is robust enough to account for the missing data [33]. For the comparisons of malaria and anaemia in the treatment groups, proportions were compared. Analyses were also done on only schoolchildren attending all visits, however, excluding visit four because only two schools at this time point were reached before the WHO declared the COVID-19 pandemic. A *p*-value less than 0.05 was considered statistically significant.

Ethical approval

The study received ethical approval from the Medical Research Coordinating Committee of Tanzania with reference number, NIMR/HQ/R.8a/Vol.IX/4120 for this study and NIMR/HQ/R.8a/Vol.IX/2818, NIMR/HQ/R.8c/Vol.I/668 for amendment, and NIMR/HQ/R.8c/Vol.I/1276 for renewal for the main study. Also, the study received authorization from the local government authorities and local school committees. Parents or guardians provided written informed consent for their children to participate, while children aged 11 or older were requested to assent. The main study also received regulatory approval from the Tanzania Medicines and Medical Devices Authority with reference number TFDA0017/CTR/0018/07.

Results

Study participants

Blood samples from 369 schoolchildren from a previous trial [10] were available for the current study. The seven primary schools visited were in Muheza District, Tanga, north-eastern Tanzania (Table 1). The study had six visits and samples from 119 participants in the control group, 134 participants in the dihydroartemisinin-piperaquine (DP) group and 116 participants in the artesunate-amodiaquine (ASAQ) group (Table 1). At baseline, the three groups had a similar distribution of sex, anaemia, bed net use, haemoglobin levels, and malaria prevalence.

Table 1 Participants' characteristics at baseline before IPTsc was initiated.

**Characteristic**	**Control (n=119)**	**DP (n=134)**	**ASAQ (n=116)**	***p value***

**Sex**				
Male, n (%)	62 (52.1)	69 (51.5)	64 (55.2)	0.83
Female, n (%)	57 (47.9)	65 (48.5)	52 (44.8)	
**Age**				
median [IQR]	10 [7 – 11]	9 [8 – 11]	10 [8 – 12]	0.44
**Age groups**				
5-9yrs, n (%)	58 (48.7)	73 (54.5)	53 (45.7)	0.37
10-15yrs, n (%)	61 (51.3)	61 (45.5)	63 (54.3)	
**Malaria prevalence by blood slide**				
Negative n (%)	86 (75.4)	89 (69.5)	77 (71.3)	0.58
Positive n (%)	28 (24.6)	39 (30.5)	31 (28.7)	
**Episodes of malaria in the last month**				
Negative, n (%)	68 (57.1)	81 (60.4)	61 (52.6)	0.46
Positive, n (%)	51 (42.9)	53 (39.6)	55 (47.4)	
**Bed net use**				
No, n (%)	18 (15.1)	32 (23.9)	27 (23.3)	0.17
Yes, n (%)	101 (84.9)	102 (76.1)	89 (76.7)	
**Haemoglobin median**				
Median [IQR]	11.7 [10.7 – 12.8]	11.7 [10.8 – 12.5]	11.5 [10.8 – 12.4]	0.45
**Anaemia**				
Anaemia, n (%)	54 (45.4)	58 (43.3)	66 (56.9)	0.08
Normal, n (%)	65 (54.6)	76 (56.7)	50 (43.1)	
**Number of Schoolchildren**				
Pangamlima, n (%)	16 (13.8)	23 (17.2)	14 (11.8)	0.99
Songa Kibaoni, n (%)	20 (17.2)	19 (14.2)	22 (18.5)	
Heinkele, n (%)	16 (13.8)	24 (17.9)	17 (14.3)	
Kwakibuyu, n (%)	16 (13.8)	18 (13.4)	17 (14.3)	
Mhamba, n (%)	18 (15.5)	18 (13.4)	16 (13.4)	
Bwitini, n (%)	13 (11.2)	14 (10.4)	13 (10.9)	
Mkulumilo, n (%)	17 (14.7)	18 (13.4)	20 (16.8)	

Breadth of malaria antigens recognition during and after the intervention phase

The breadth of the six-malaria antigens recognition was compared between schoolchildren from the three study groups, DP, ASAQ, and the control, at baseline and across all study visits. The number of malaria antigens recognised significantly increased during the study period in all three groups and there was no significant difference in the trend of the breath of malaria antigen recognition between the study groups (Trend test across ordered groups: DP, z-score = 5.92, $p < 0.001$, ASAQ, z-score = 6.64, $p < 0.001$ and control, z-score = 5.85, $p < 0.001$) during and after the intervention phase (Fig 1). Compared with the control group, from baseline the DP group had non-significantly lower difference in mean change in breadth of malaria antigen recognition in all study visits (Tabel 2), and the ASAQ group had non-significantly higher difference in mean change in breadth of malaria antigen recognition (Table 3).

Fig 1. Changes in the breadth of malaria antigen recognition (means with 95%CI) across six study visits four months apart. In year-one, visit 1 to visit 3 dihydroartemisinin-piperaquine (DP) and artesunate-amodiaquine (ASAQ) were given to schoolchildren, and in year-two visit 4 to visit 6 no study drugs were given.

Table 2 The impact of intermittent preventive treatment with dihydroartemisinin-piperaquine on the breadth of malaria antigen recognition.

Study visit	N	Mean (Control)	N	Mean (DP)	Difference in mean change in breadth of malaria antigen recognition from baseline (95% CI)	*p value*
Visit 1 - Month 0 (Baseline)	60	2.27	71	2.47	--	
Visit 2 - Month 4	115	3.42	130	3.12	-0.50 (-1.22 – 0.22)	0.18
Visit 3 - Month 8	107	3.00	126	2.52	-0.68 (-1.41 – 0.05)	0.07
Visit 4 - Month 12	29	2.52	39	2.51	-0.20 (-1.21 – 0.80)	0.69
Visit 5 - Month 16	110	3.66	131	3.83	-0.03 (-0.75 – 0.70)	0.94
Visit 6 - Month 20	115	4.09	131	3.60	-0.69 (-1.41 – 0.03)	0.06

Table 3 The impact of intermittent preventive treatment with artesunate-amodiaquine on the breadth of malaria antigen recognition.

Study visit	N	Mean (Control)	N	Mean (ASAQ)	Difference in mean change in breadth of malaria antigen recognition from baseline (95% CI)	*p value*
Visit 1 - Month 0 (Baseline)	60	2.27	61	1.95	--	
Visit 2 - Month 4	115	3.42	112	3.28	0.18 (-0.57 – 0.92)	0.65
Visit 3 - Month 8	107	3.00	107	3.19	0.50 (-0.25 – 1.26)	0.19
Visit 4 - Month 12	29	2.52	33	2.58	0.37 (-0.67 – 1.42)	0.48
Visit 5 - Month 16	110	3.66	111	3.74	0.39 (-0.36 – 1.14)	0.31
Visit 6 - Month 20	115	4.09	111	4.03	0.26 (-0.49 – 1.01)	0.50

IgG reactivity to GLURP-R2, MSP1, and MSP3

The IgG reactivity was analysed longitudinally in the DP and ASAQ groups. The changes in mean IgG responses against GLURP-R2, MSP1, and MSP3 in the ASAQ group were not significantly different from the control group across all study visits (Figs 2a, 2b, 2c, S1 Table). In the DP group, however, IgG reactivity to GLURP-R2 was significantly lower at all follow-up study visits (Fig 2a and S1 Table) and lower against MSP3 at visit six (mean OD -0.06 [95% CI -0.12 – -0.00] $p = 0.039$) (S1 Table and Fig 2c). The IgG reactivity to MSP1 in

the DP group was not significantly different from the control group in either analysis (Fig 2b, S1 Table).

Fig 2. Antibody trends against *P. falciparum* after intermittent preventive treatment for malaria in school-aged children (IPTsc). The study drugs, dihydroartemisinin-piperaquine (DP) and artesunate amodiaquine (ASAQ) were given from visit 1 to visit 3 then during follow-up visits from visit 4 to visit 6 no study drugs were given to all participants: (**a)** antibodies trend against the glutamate rich protein-region 2 (GLURP-R2), **(b)** antibodies trend against the merozoite surface protein 1 (MSP1), **(c)** antibodies trend against the merozoite surface protein 3 (MSP3), **(d, e, f)** antibodies trend against three cysteine-rich interdomain region (CIDR) domains (CIDRα1.1, CIDRα1.4 and CIDRα1.5) of *P. falciparum* erythrocyte membrane protein 1 (PfEMP1).

IgG reactivity to PfEMP1 domains (CIDRa1.1, CIDRa1.4 and CIDRa1.5)

The longitudinal analysis of IgG reactivity to the three domains of PfEMP1, CIDRa1.1, CIDRa1.4 and CIDRa1.5 showed no significant differences in change in reactivity between the DP and ASAQ groups as compared to the control group during the first 16 months of the study (Figs 2d-f, S1 Table). However, at the last visit, visit six, mean IgG reactivity to CIDRa1.4 and CIDRa1.5 in the DP group were significantly lower as compared to the control group (mean OD -0.20 [95% CI -0.32 – -0.08] $p = 0.001$ and mean OD -0.10 [95% CI -0.20 – 0.00] $p = 0.05$, respectively) (Figs 2e and 2f).

IgG reactivity in participants attending all visits

When analysing only schoolchildren attending all visits, and excluding visit four, n = 60, 52 and 49 in the DP, ASAQ and control groups, respectively, showed similar outcomes (S1 Fig and S2 Table). However, in the DP group, the change in mean IgG levels against MSP3 was also significantly lower at visit five (mean OD -0.10 [95% CI -0.17 – -0.02] $p = 0.010$), and at visit six (mean OD -0.08 [95% CI -0.15 – -0.01] $p = 0.033$) and the differences in mean changes of IgG reactivity to CIDRa1.4 and CIDRa1.5 were not significant.

Haemoglobin concentration, anaemia and malaria prevalence

The mean haemoglobin levels were significantly higher at visit four in the DP and ASAQ groups as compared to the control group and ((0.93 g/dL [95% CI 0.40 – 1.46] $p = 0.001$) and (0.98 g/dL [95% CI 0.44 – 1.54] $p < 0.001$), respectively) (S2a Fig). The prevalence of anaemia was lower in the DP and ASAQ groups at visit four as compared with the control group, though not significant (-22.86 percentage points [95% CI -45.65 –0.01] $p = 0.06$, and -18.67 percentage points [95% CI -40.50 – 3.16] $p = 0.10$, respectively). Lastly, the malaria prevalence was lower in both the DP and ASAQ group at visit four, but only significant in the DP group (-27.14 percentage points [95% CI -44.69 – 9.59] $p = 0.003$) (ASAQ group: -19.13 percentage points [95% CI -39.6 – 0.79] $p = 0.69$), as compared to the control group. At visit six there was no difference in change of haemoglobin concentration, anaemia or malaria prevalence between the study groups and the control group as also seen in the larger trial study by [10].

Discussion

In a recently completed trial, IPTsc with DP and ASAQ was proven to be a feasible strategy to reduce malaria burden and related morbidities in schoolchildren in highly malaria-endemic areas of north-eastern Tanzania [10, 34]. This study evaluated the IgG reactivity to a panel of

six malaria antigens associated with the exposure and protection of malaria [21, 22, 35, 36] during and up to one year after the last intervention to assess whether IPTsc with either DP or ASAQ interferes with the natural immune response to these six antigens. The breadth of antibody response was shown to increase in both intervention groups during the study period, comparable to the control group, suggesting that all schoolchildren are exposed to malaria parasites sufficiently enough to generate antibodies against the tested antigens. In particular, the schoolchildren in the ASAQ group and in DP group showed no difference in the breath of malaria antigen recognition from the control group across all study visits and no differences in the change of antibody levels for all individual antigens during and after the intervention period. Schoolchildren in the DP group, however, had a significantly lower antibody response to some of the tested antigens, and in particular antibody responses to GLURP-R2 was significantly lower in all study visit except in the fourth visit at month 12, when antibodies responses were non-significantly lower. In the DP group, antibody responses to MSP3, CIDRa1.4 and CIDRa1.5 were lower at month 20 as well. The lower antibody reactivity and the significant reduction of malaria prevalence at visit four, the first visit after the intervention period, observed in the DP group is consistent with the effectiveness of the parasite clearance by the piperaquine component of the regimen owing to its long half-life [37]. These findings are consistent with findings from the main trial study [10] where the prevalence of parasitaemia reduction was significantly higher in the DP group as compared to the ASAQ group at visit four. At visit six, there were no differences in the prevalence of parasitaemia between the groups [34], which indicates that the relatively lower levels of antibodies to GLURP-R2 and MSP3 in the DP group one year after the intervention were not associated with lower immunity to malaria.

To our knowledge, this is the first study to analyse the impact of IPTsc with DP or ASAQ on the antibody response to six antigens of *P. falciparum* and demonstrates that the development

of the immune response to *P. falciparum* antigens remains mostly unaffected by the interventions. These observations are consistent with findings from an IPTi study with SP in Mozambique [16, 38] and a SMC study with SP plus AQ in Mali [39, 40]. These results suggest that IPTsc with DP and ASAQ is an effective strategy to reduce the malaria burden without interfering with the development of IgG against malaria antigens in areas with high malaria incidence. Future studies may explore whether this holds true in low malaria-endemic areas as well. The current study has several limitations including not all participants from the main trial study were included, so the effect of IPTsc on the immune response of the entire cohort was not examined. Visit four, the first visit of the non-intervention phase, included two schools only due to the COVID-19 pandemic. Furthermore, this study investigated only six malaria antigens where naturally acquired immunity to malaria seems to target multiple antigens and stages of the parasite [41, 42], though antibodies to the tested antigens are associated with protection from severe malaria [21, 43, 44], seem to be important in controlling infections [23-26], and are targets for malaria vaccine development [45-48].

Conclusions

We have shown that effective IPTsc with either DP or ASAQ does not interfere with the development of antibodies to different malaria antigens of the blood stages, expressed on the surface of infected erythrocytes and on merozoites and important targets of the naturally acquired immunity. Future studies may explore a wider range of malaria antigens using a much longer IPTsc programme.

Acknowledgements

The authors thank all study participants and staff who worked in the clinical trial. We thank staff from all organisations including the National Institute for Medical Research and the collaborating Ministries of the Government of Tanzania, who made this study possible. We

thank Professor Michael Theisen (University of Copenhagen), for supplying the MSP1, MSP3, and GLURP-R2 domains.

Author Contributions

Eric Lyimo: Conceptualization, Data Curation, Formal Analysis, Investigation, Methodology, Resources, Validation, Visualization, Writing – Original Draft**. Geofrey Makenga:** Conceptualization, Data Curation, Formal Analysis, Funding Acquisition, Investigation, Project Administration, Resources, Supervision, Writing – Review & Editing· **Louise Turner:** Methodology, Resources, Writing – Review & Editing **Thomas Lavstsen:** Methodology, Resources, Writing – Review & Editing. **John P. A. Lusingu**: Funding Acquisition, Investigation, Project Administration, Resources, Supervision, Writing – Review & Editing. **Jean-Pierre Van geertruyden** Funding Acquisition, Investigation, Project Administration, Resources, Supervision, Writing – Review & Editing. **Daniel T. R. Minja:** Data Curation, Funding Acquisition, Investigation, Project Administration, Resources, Supervision, Writing – Review & Editing. **Christian W. Wang:** Conceptualization, Formal Analysis, Funding Acquisition, Investigation, Methodology, Resources, Supervision, Visualization, Writing – Review & Editing. **Vito Baraka:** Funding Acquisition, Investigation, Project Administration, Resources, Supervision, Validation, Writing – Review & Editing

Funding

The clinical trial was funded by the Flemish Interuniversity Council (VLIRUOS), Belgium (TEAM initiative, grant number TZ2017TEA451A102). This study was funded by the Malaria Research and Capacity Building for field trials in Tanzania (MaReCa) project as part of the EDCTP2 programme supported by the European Union (Grant number TMA2015SF–998-MaReCa awarded to JPAL).

References

1. WHO. World malaria report: World Health Organization; 2023.

2. WHO. WHO Policy Recommendation: Seasonal Malaria Chemoprevention (SMC) for Plasmodium falciparum malaria control in highly seasonal transmission areas of the Sahel sub-region in Africa. World Health Organization, 2012.

3. WHO. Guidelines for malaria, 14 March 2023. Geneva: World Health Organization, 2023 Contract No.: WHO/UCN/GMP/2023.01.

4. Kigozi SP, Kigozi RN, Epstein A, Mpimbaza A, Sserwanga A, Yeka A, et al. Rapid shifts in the age-specific burden of malaria following successful control interventions in four regions of Uganda. Malar J. 2020;19(1):128. PMID: 32228584; PMCID: PMC7106889. doi: 10.1186/s12936-020-03196-7.

5. Nankabirwa JI, Wandera B, Amuge P, Kiwanuka N, Dorsey G, Rosenthal PJ, et al. Impact of intermittent preventive treatment with dihydroartemisinin-piperaquine on malaria in Ugandan schoolchildren: a randomized, placebo-controlled trial. Clin Infect Dis. 2014;58(10):1404-12. PMID: 24621953; PMCID: PMC4001293. doi: 10.1093/cid/ciu150.

6. Cohee LM, Opondo C, Clarke SE, Halliday KE, Cano J, Shipper AG, et al. Preventive malaria treatment among school-aged children in sub-Saharan Africa: a systematic review and meta-analyses. Lancet Glob Health. 2020;8(12):e1499-e511. PMID: 33222799; PMCID: PMC7721819. doi: 10.1016/S2214-109X(20)30325-9.

7. Makenga G, Menon S, Baraka V, Minja DTR, Nakato S, Delgado-Ratto C, et al. Prevalence of malaria parasitaemia in school-aged children and pregnant women in endemic settings of sub-Saharan Africa: A systematic review and meta-analysis. Parasite Epidemiol Control. 2020;11:e00188. PMID: 33145445; PMCID: PMC7591779. doi: 10.1016/j.parepi.2020.e00188.

8. Matangila JR, Mitashi P, Inocencio da Luz RA, Lutumba PT, Van Geertruyden JP. Efficacy and safety of intermittent preventive treatment for malaria in schoolchildren: a systematic review. Malar J. 2015;14:450. PMID: 26574017; PMCID: PMC4647321. doi: 10.1186/s12936-015-0988-5.

9. Matangila JR, Doua JY, Mitashi P, da Luz RI, Lutumba P, Van Geertruyden JP. Efficacy and safety of intermittent preventive treatment in schoolchildren with sulfadoxine/pyrimethamine (SP) and SP plus piperaquine in Democratic Republic of the Congo: a randomised controlled trial. Int J Antimicrob Agents. 2017;49(3):339-47. PMID: 28108368. doi: 10.1016/j.ijantimicag.2016.11.017.

10. Makenga G, Baraka V, Francis F, Nakato S, Gesase S, Mtove G, et al. Effectiveness and safety of intermittent preventive treatment with dihydroartemisinin-piperaquine or artesunate-amodiaquine for reducing malaria and related morbidities in schoolchildren in Tanzania: a randomised controlled trial. Lancet Glob Health. 2023;11(8):e1277-e89. PMID: 37474234. doi: 10.1016/S2214-109X(23)00204-8.

11. Kobbe R, Hogan B, Adjei S, Klein P, Kreuels B, Loag W, et al. Follow-up survey of children who received sulfadoxine-pyrimethamine for intermittent preventive antimalarial treatment in infants. J Infect Dis. 2011;203(4):556-60. PMID: 21248056; PMCID: PMC3071230. doi: 10.1093/infdis/jiq079.

12. Greenwood BM, David PH, Otoo-Forbes LN, Allen SJ, Alonso PL, Armstrong Schellenberg JR, et al. Mortality and morbidity from malaria after stopping malaria chemoprophylaxis. Trans R Soc Trop Med Hyg. 1995;89(6):629-33. PMID: 8594677. doi: 10.1016/0035-9203(95)90419-0.

13. Djontu JC, Lloyd YM, Megnekou R, Seumko'o RMN, Salanti A, Taylor DW, et al. Antibodies to full-length and the DBL5 domain of VAR2CSA in pregnant women after long-term implementation of intermittent preventive treatment in Etoudi, Cameroon. PLoS One.

2020;15(8):e0237671. PMID: 32797068; PMCID: PMC7428160. doi: 10.1371/journal.pone.0237671.

14. Schreiber N, Kobbe R, Adjei S, Adjei O, Klinkert MQ, May J. Immune responses after single-dose sulphadoxine-pyrimethamine indicate underestimation of protective efficacy of intermittent preventive treatment in infants. Trop Med Int Health. 2007;12(10):1157-63. PMID: 17956497. doi: 10.1111/j.1365-3156.2007.01902.x.

15. Boulanger D, Sarr JB, Fillol F, Sokhna C, Cisse B, Schacht AM, et al. Immunological consequences of intermittent preventive treatment against malaria in Senegalese preschool children. Malar J. 2010;9:363. PMID: 21167018; PMCID: PMC3012051. doi: 10.1186/1475-2875-9-363.

16. Quelhas D, Jimenez A, Quinto L, Serra-Casas E, Mayor A, Cistero P, et al. IgG against Plasmodium falciparum variant surface antigens and growth inhibitory antibodies in Mozambican children receiving intermittent preventive treatment with sulfadoxine-pyrimethamine. Immunobiology. 2011;216(7):793-802. PMID: 21257227. doi: 10.1016/j.imbio.2010.12.010.

17. Baraka V, Ishengoma DS, Fransis F, Minja DT, Madebe RA, Ngatunga D, et al. High-level Plasmodium falciparum sulfadoxine-pyrimethamine resistance with the concomitant occurrence of septuple haplotype in Tanzania. Malar J. 2015;14:439. PMID: 26542942; PMCID: PMC4635599. doi: 10.1186/s12936-015-0977-8.

18. Bwire GM, Mikomangwa WP, Kilonzi M. Occurrence of septuple and elevated Pfdhfr-Pfdhps quintuple mutations in a general population threatens the use of sulfadoxine-pyrimethamine for malaria prevention during pregnancy in eastern-coast of Tanzania. BMC Infect Dis. 2020;20(1):530. PMID: 32698764; PMCID: PMC7374904. doi: 10.1186/s12879-020-05253-7.

19. Turner L, Lavstsen T, Berger SS, Wang CW, Petersen JE, Avril M, et al. Severe malaria is associated with parasite binding to endothelial protein C receptor. Nature. 2013;498(7455):502-5. PMID: 23739325; PMCID: PMC3870021. doi: 10.1038/nature12216.

20. Mkumbaye SI, Wang CW, Lyimo E, Jespersen JS, Manjurano A, Mosha J, et al. The Severity of Plasmodium falciparum Infection Is Associated with Transcript Levels of var Genes Encoding Endothelial Protein C Receptor-Binding P. falciparum Erythrocyte Membrane Protein 1. Infection and immunity. 2017;85(4). PMID: 28138022; PMCID: PMC5364309. doi: 10.1128/IAI.00841-16.

21. Obeng-Adjei N, Larremore DB, Turner L, Ongoiba A, Li S, Doumbo S, et al. Longitudinal analysis of naturally acquired PfEMP1 CIDR domain variant antibodies identifies associations with malaria protection. JCI insight. 2020;5(12). PMID: 32427581; PMCID: PMC7406271. doi: 10.1172/jci.insight.137262.

22. Turner L, Lavstsen T, Mmbando BP, Wang CW, Magistrado PA, Vestergaard LS, et al. IgG antibodies to endothelial protein C receptor-binding cysteine-rich interdomain region domains of Plasmodium falciparum erythrocyte membrane protein 1 are acquired early in life in individuals exposed to malaria. Infection and immunity. 2015;83(8):3096-103. PMID: 26015475; PMCID: PMC4496620. doi: 10.1128/IAI.00271-15.

23. Adamou R, Dechavanne C, Sadissou I, d'Almeida T, Bouraima A, Sonon P, et al. Plasmodium falciparum merozoite surface antigen-specific cytophilic IgG and control of malaria infection in a Beninese birth cohort. Malar J. 2019;18(1):194. PMID: 31185998; PMCID: PMC6560827. doi: 10.1186/s12936-019-2831-x.

24. Adu B, Cherif MK, Bosomprah S, Diarra A, Arthur FK, Dickson EK, et al. Antibody levels against GLURP R2, MSP1 block 2 hybrid and AS202.11 and the risk of malaria in

children living in hyperendemic (Burkina Faso) and hypo-endemic (Ghana) areas. Malar J. 2016;15:123. PMID: 26921176; PMCID: PMC4769494. doi: 10.1186/s12936-016-1146-4.

25. Lusingu JP, Vestergaard LS, Alifrangis M, Mmbando BP, Theisen M, Kitua AY, et al. Cytophilic antibodies to Plasmodium falciparum glutamate rich protein are associated with malaria protection in an area of holoendemic transmission. Malar J. 2005;4:48. PMID: 16194274; PMCID: PMC1262760. doi: 10.1186/1475-2875-4-48.

26. Hamre KES, Ondigo BN, Hodges JS, Dutta S, Theisen M, Ayodo G, et al. Antibody Correlates of Protection from Clinical Plasmodium falciparum Malaria in an Area of Low and Unstable Malaria Transmission. Am J Trop Med Hyg. 2020;103(6):2174-82. PMID: 33124533; PMCID: PMC7695051. doi: 10.4269/ajtmh.18-0805.

27. Makenga G, Baraka V, Francis F, Nakato S, Gesase S, Mtove G, et al. Effectiveness and safety of intermittent preventive treatment for malaria using either dihydroartemisinin-piperaquine or artesunate-amodiaquine in reducing malaria related morbidities and improving cognitive ability in school-aged children in Tanzania: A study protocol for a controlled randomised trial. Contemp Clin Trials Commun. 2020;17:100546. PMID: 32382685; PMCID: PMC7201189. doi: 10.1016/j.conctc.2020.100546.

28. Mtove G, Mugasa JP, Messenger LA, Malima RC, Mangesho P, Magogo F, et al. The effectiveness of non-pyrethroid insecticide-treated durable wall lining to control malaria in rural Tanzania: study protocol for a two-armed cluster randomized trial. BMC Public Health. 2016;16:633. PMID: 27456339; PMCID: PMC4960851. doi: 10.1186/s12889-016-3287-3.

29. Lyimo E, Fougeroux C, Malabeja A, Mbwana J, Hayuma PM, Liheluka E, et al. Seroprevalence of SARS-CoV-2 antibodies among children and adolescents recruited in a malariometric survey in north-eastern Tanzania July 2021. BMC Infect Dis. 2022;22(1):846. PMID: 36371172; PMCID: PMC9652923. doi: 10.1186/s12879-022-07820-6.

30. Cham GK, Kurtis J, Lusingu J, Theander TG, Jensen AT, Turner L. A semi-automated multiplex high-throughput assay for measuring IgG antibodies against Plasmodium falciparum erythrocyte membrane protein 1 (PfEMP1) domains in small volumes of plasma. Malar J. 2008;7:108. PMID: 18549480; PMCID: PMC2435541. doi: 10.1186/1475-2875-7-108.

31. Corran PH, Cook J, Lynch C, Leendertse H, Manjurano A, Griffin J, et al. Dried blood spots as a source of anti-malarial antibodies for epidemiological studies. Malar J. 2008;7(1):195. PMID: 18826573; PMCID: PMC2567984. doi: 10.1186/1475-2875-7-195.

32. Lyimo E, Haslund LE, Ramsing T, Wang CW, Efunshile AM, Manjurano A, et al. In Vivo Imaging of the Buccal Mucosa Shows Loss of the Endothelial Glycocalyx and Perivascular Hemorrhages in Pediatric Plasmodium falciparum Malaria. Infection and immunity. 2020;88(3). PMID: 31871101; PMCID: PMC7035942. doi: 10.1128/IAI.00679-19.

33. Peters SA, Bots ML, den Ruijter HM, Palmer MK, Grobbee DE, Crouse JR, 3rd, et al. Multiple imputation of missing repeated outcome measurements did not add to linear mixed-effects models. J Clin Epidemiol. 2012;65(6):686-95. PMID: 22459429. doi: 10.1016/j.jclinepi.2011.11.012.

34. Makenga G, Seth MD, Baraka V, Mmbando BP, Challe DP, Francis F, et al. Implementation research of a cluster randomized trial evaluating the implementation and effectiveness of intermittent preventive treatment for malaria using dihydroartemisinin-piperaquine on reducing malaria burden in school-aged children in Tanzania: methodology, challenges, and mitigation. Malar J. 2023;22(1):7. PMID: 36609279; PMCID: PMC9816525. doi: 10.1186/s12936-022-04428-8.

35. Courtin D, Oesterholt M, Huismans H, Kusi K, Milet J, Badaut C, et al. The quantity and quality of African children's IgG responses to merozoite surface antigens reflect

protection against Plasmodium falciparum malaria. PLoS One. 2009;4(10):e7590. PMID: 19859562; PMCID: PMC2763201. doi: 10.1371/journal.pone.0007590.

36. Kana IH, Singh SK, Garcia-Senosiain A, Dodoo D, Singh S, Adu B, et al. Breadth of Functional Antibodies Is Associated With Plasmodium falciparum Merozoite Phagocytosis and Protection Against Febrile Malaria. J Infect Dis. 2019;220(2):275-84. PMID: 30820557. doi: 10.1093/infdis/jiz088.

37. Tarning J, Ashley EA, Lindegardh N, Stepniewska K, Phaiphun L, Day NP, et al. Population pharmacokinetics of piperaquine after two different treatment regimens with dihydroartemisinin-piperaquine in patients with Plasmodium falciparum malaria in Thailand. Antimicrob Agents Chemother. 2008;52(3):1052-61. PMID: 18180343; PMCID: PMC2258541. doi: 10.1128/AAC.00955-07.

38. Quelhas D, Puyol L, Quinto L, Serra-Casas E, Nhampossa T, Macete E, et al. Impact of intermittent preventive treatment with sulfadoxine-pyrimethamine on antibody responses to erythrocytic-stage Plasmodium falciparum antigens in infants in Mozambique. Clinical and vaccine immunology : CVI. 2008;15(8):1282-91. PMID: 18495848; PMCID: PMC2519318. doi: 10.1128/CVI.00044-08.

39. Mahamar A, Issiaka D, Barry A, Attaher O, Dembele AB, Traore T, et al. Effect of seasonal malaria chemoprevention on the acquisition of antibodies to Plasmodium falciparum antigens in Ouelessebougou, Mali. Malar J. 2017;16(1):289. PMID: 28720100; PMCID: PMC5516340. doi: 10.1186/s12936-017-1935-4.

40. Mahamar A, Issiaka D, Youssouf A, Niambele SM, Soumare HM, Attaher O, et al. Effect of 4 years of seasonal malaria chemoprevention on the acquisition of antibodies to Plasmodium falciparum antigens in Ouelessebougou, Mali. Malar J. 2021;20(1):23. PMID: 33413417; PMCID: PMC7788529. doi: 10.1186/s12936-020-03542-9.

41. Gonzales SJ, Reyes RA, Braddom AE, Batugedara G, Bol S, Bunnik EM. Naturally Acquired Humoral Immunity Against Plasmodium falciparum Malaria. Front Immunol. 2020;11:594653. PMID: 33193447; PMCID: PMC7658415. doi: 10.3389/fimmu.2020.594653.

42. Healer J, Chiu CY, Hansen DS. Mechanisms of naturally acquired immunity to P. falciparum and approaches to identify merozoite antigen targets. Parasitology. 2018;145(7):839-47. PMID: 29144217. doi: 10.1017/S0031182017001949.

43. Hviid L, Lavstsen T, Jensen AT. A vaccine targeted specifically to prevent cerebral malaria - is there hope? Expert Rev Vaccines. 2018;17(7):565-7. PMID: 29898617. doi: 10.1080/14760584.2018.1488591.

44. Tessema SK, Nakajima R, Jasinskas A, Monk SL, Lekieffre L, Lin E, et al. Protective Immunity against Severe Malaria in Children Is Associated with a Limited Repertoire of Antibodies to Conserved PfEMP1 Variants. Cell Host Microbe. 2019;26(5):579-90 e5. PMID: 31726028. doi: 10.1016/j.chom.2019.10.012.

45. Theisen M, Adu B, Mordmuller B, Singh S. The GMZ2 malaria vaccine: from concept to efficacy in humans. Expert Rev Vaccines. 2017;16(9):907-17. PMID: 28699823. doi: 10.1080/14760584.2017.1355246.

46. Dassah S, Adu B, Tiendrebeogo RW, Singh SK, Arthur FKN, Sirima SB, et al. GMZ2 Vaccine-Induced Antibody Responses, Naturally Acquired Immunity and the Incidence of Malaria in Burkinabe Children. Front Immunol. 2022;13:899223. PMID: 35720297; PMCID: PMC9200992. doi: 10.3389/fimmu.2022.899223.

47. Tiono AB, Plieskatt JL, Ouedraogo A, Soulama BI, Miura K, Bougouma EC, et al. A randomized first-in-human phase I trial of differentially adjuvanted Pfs48/45 malaria vaccines in Burkinabe adults. J Clin Invest. 2024;134(7). PMID: 38290009; PMCID: PMC10977980. doi: 10.1172/JCI175707.

48. Amoah LE, Nuvor SV, Obboh EK, Acquah FK, Asare K, Singh SK, et al. Natural antibody responses to Plasmodium falciparum MSP3 and GLURP(R0) antigens are associated with low parasite densities in malaria patients living in the Central Region of Ghana. Parasit Vectors. 2017;10(1):395. PMID: 28835262; PMCID: PMC5569498. doi: 10.1186/s13071-017-2338-7.

Supporting information

S1 Fig. Antibody trends against *P. falciparum* after intermittent preventive treatment for malaria in school-aged children (IPTsc). Figures showing participants who attended all visits, and visit 4 has been excluded in the analysis: **(a)** antibodies trend against the glutamate rich protein-region 2 (GLURP-R2), **(b)** antibodies trend against the merozoite surface protein 1 (MSP1), **(c)** antibodies trend against the merozoite surface protein 3 (MSP3), **(d, e, f)** antibodies trend against three cysteine-rich interdomain region (CIDR) domains (CIDRα1.1, CIDRα1.4 and CIDRα1.5) of *P. falciparum* erythrocyte membrane protein 1 (PfEMP1).

S2 Fig. The impact of intermittent preventive treatment on malaria in school-aged children (IPTsc) on **(a)** change of mean haemoglobin, **(b)** anaemia prevalence and **(c)** malaria prevalence from baseline, at each visit per study group (n=369).

S1 Table. Linear mixed effect analysis on the impact of IPTsc intervention on antibody response against six *P. falciparum* antigens in schoolchildren aged between 5 – 15 years, n=369.

S2 Table. Linear mixed effect analysis on the impact of IPTsc intervention on antibody response against six *P. falciparum* antigens in schoolchildren aged between 5 – 15 years. Analysis based on participants who attended all visits, and visit 4 has been excluded in the analysis, n = 161.

Click here to access/download;Figure;Fig1.TIF

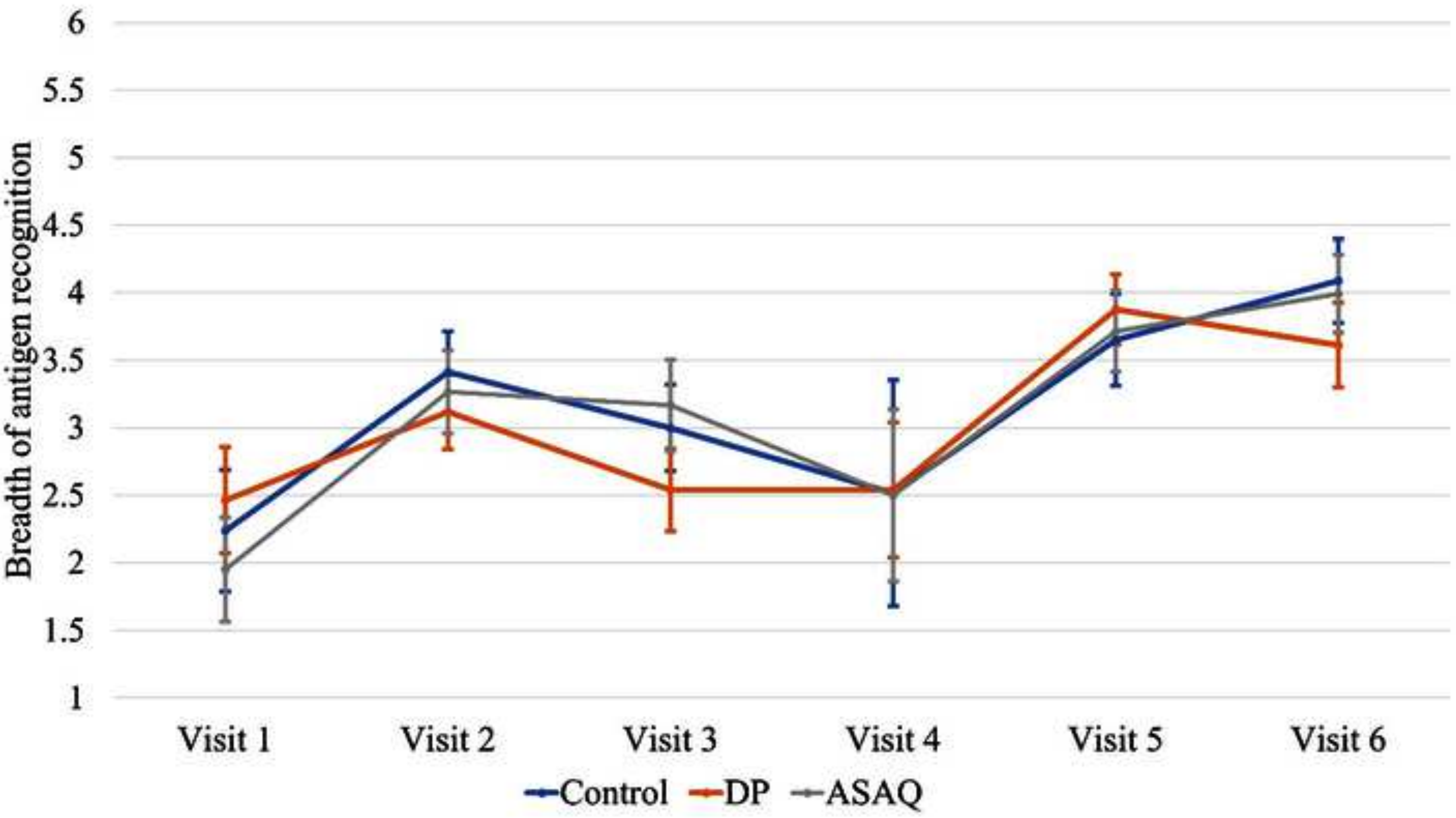

Fig2

Click here to access/download;Figure;Fig2.TIF

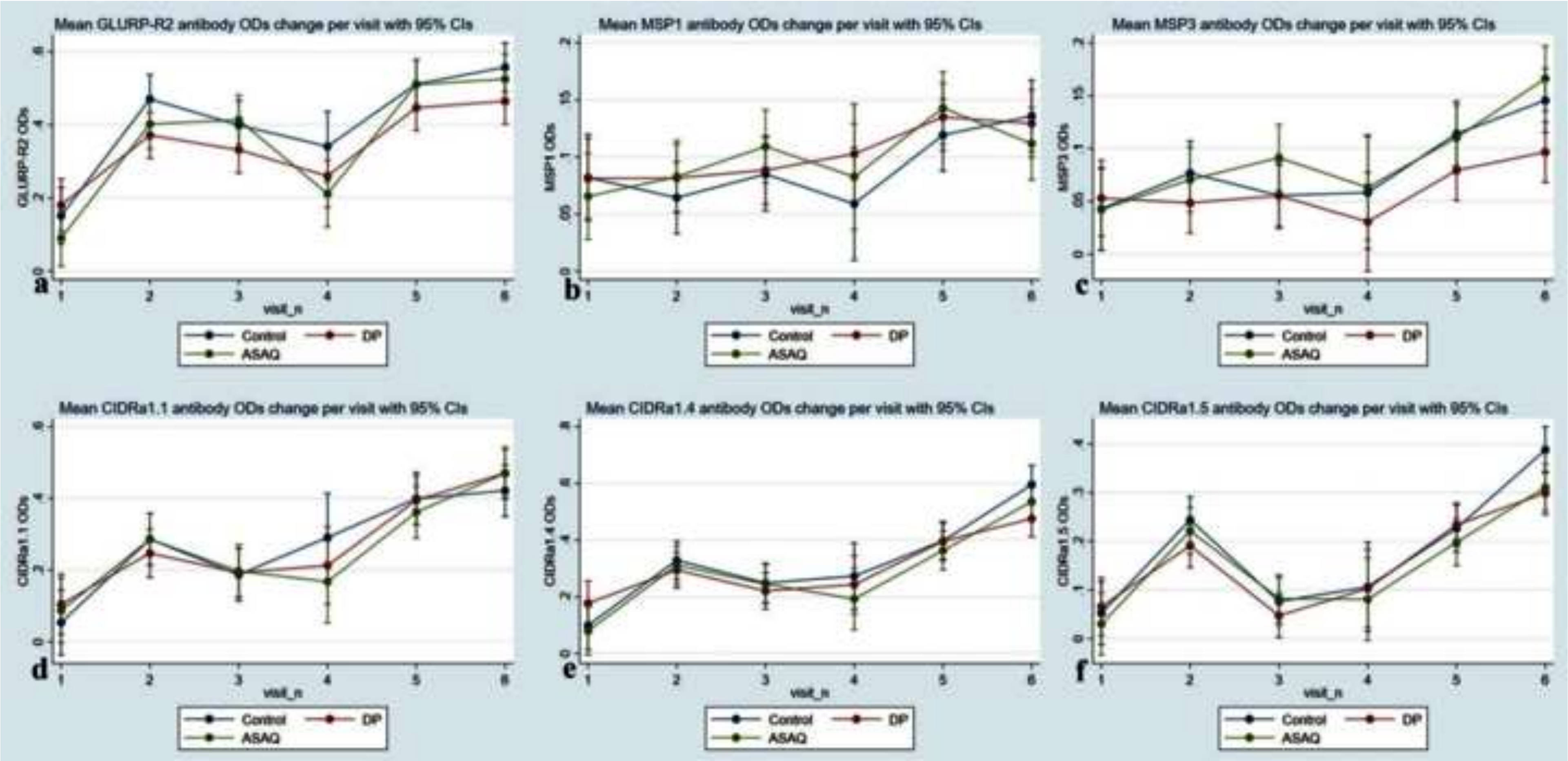

S1_Fig

Click here to access/download;Figure;S1_Fig.TIF

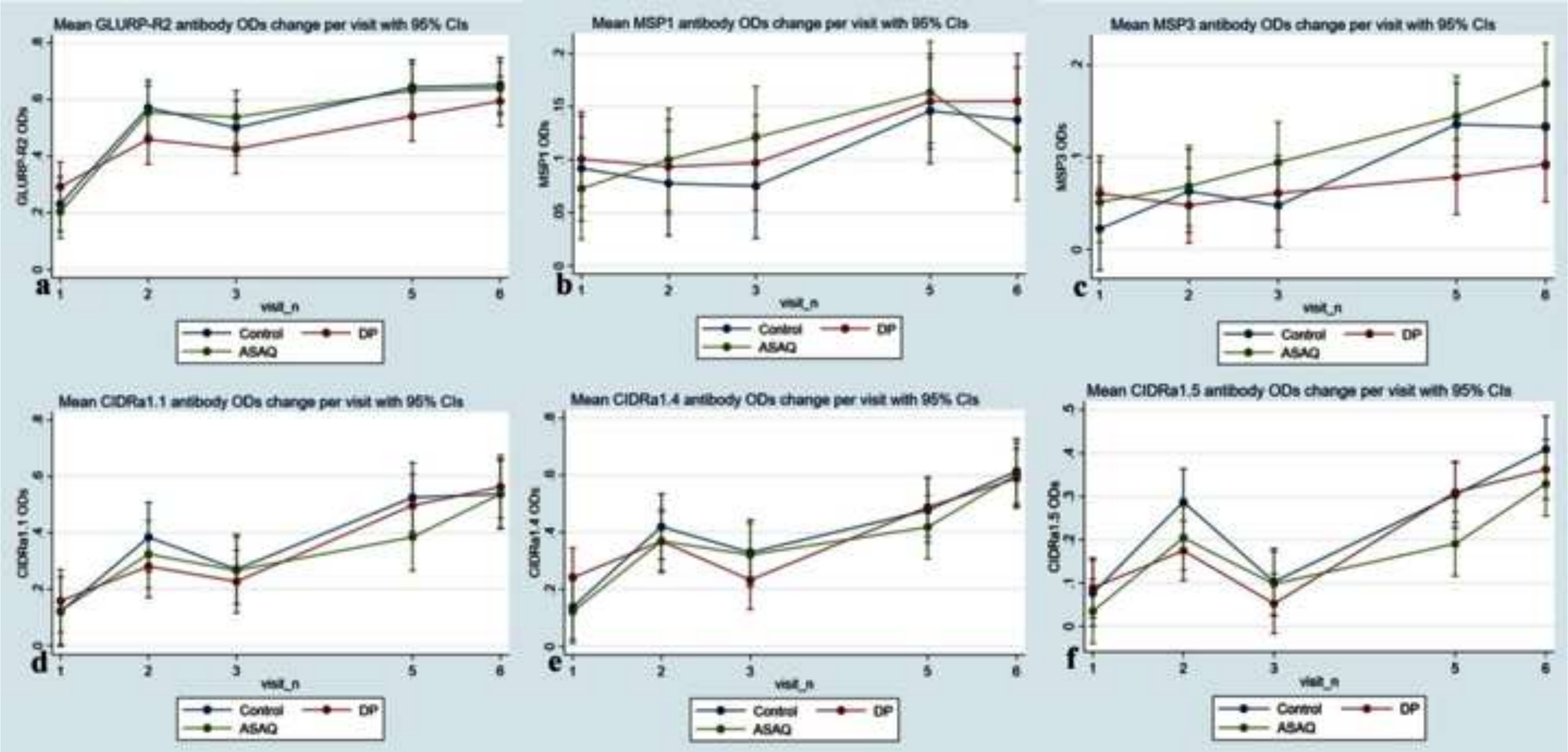

S2_Fig

Click here to access/download;Figure;S2_Fig.TIF

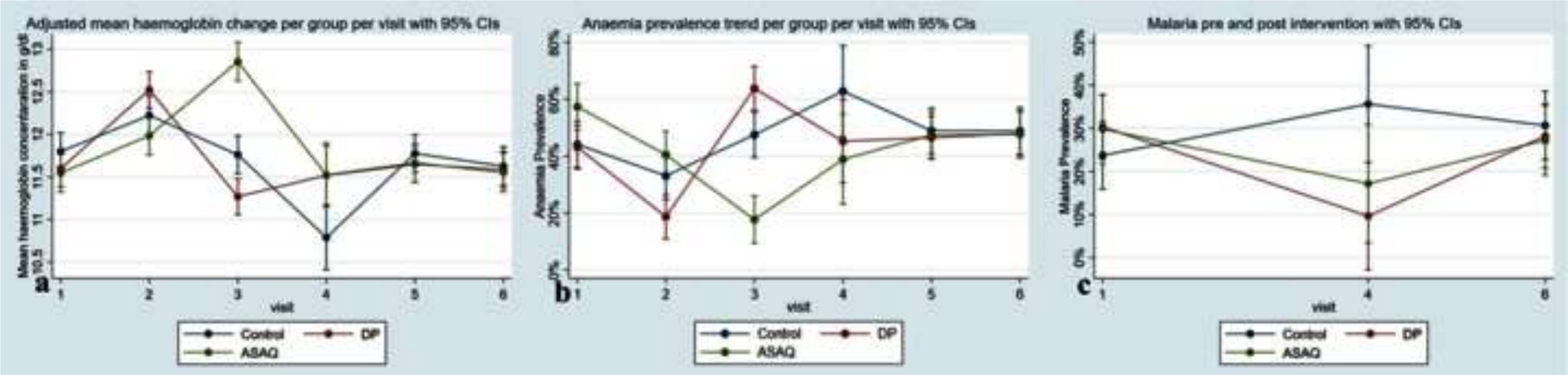

Click here to access/download
Supporting Information
Supporting information.docx
