# Supplementary material for: The impact of intermittent preventive treatment in school aged children with dihydroartemisinin piperaquine and artesunate amodiaquine on IgG response against six blood stage Plasmodium falciparum antigens
Source: PLoS One. 2025 Jan 30;20(1):e0316482. doi: 10.1371/journal.pone.0316482 (PMC11781616; doi:10.1371/journal.pone.0316482)
Supplement: Attachment — Submitted filename: REVIEWER COMMENTS.docx [file pone.0316482.s006.docx]

Dear Editor,

Thank you for finding me worthy as a reviewer for the manuscript PONE-D-24-14848.

**General**: The manuscript presents immunologic information on IgG responses to six blood stage *Plasmodium falciparum* antigens following Intermittent Preventive Treatment in school aged children (IPTsc) with Dihydroartemisinin Piperaquine (DP) and Artesunate Amodiaquine (ASAQ). The **Title** is apt while the **Abstract** clearly summarizes the study.

The manuscript describes a technically sound piece of research based on scientific concepts and acceptable methodology. The conclusions are based on and supported by the data presented. However, there are some aspects that need strengthening. Please see the uploaded reviewed manuscript with track changes (**PONE-D-24-14848 [Rev.]**) for detailed comments/suggestions/corrections on all sections of the manuscript, a few of which are given below.

**English Language Quality**: Above average but requires editing to expiate for the many typos and grammatical infelicities.

**Study design, site and participants:** The authors need to improve on the English language quality in describing the study design. There were ambiguities in the use of **”This study”** under study design, site and participants (Please refer to Lines 100, 109) and elsewhere in the manuscript. Succeeding a foregoing sentence with **”This study”** connotes reference to the earlier sentence. Better to use **”This present study”** for their study to differentiate it from the earlier study trial.

**Lines 59-60**: Delete the words **Intermittent preventive treatment of malaria in school-aged children** as indicated here and subsequently, having been fully mentioned earlier in lines 53-54.

**Lines 147/148**: The first sentence should be deleted as indicated.

**Lines 157-160**: The highlighted sentence is vague and needs to be recast for better comprehension.

**Lines 201-204**: The sentence should be deleted as indicated.

**Lines 204-205**: The sentence should be recast/rephrased as indicated.

**Lines 283**: Change study group and the control groups to "**treatment and control groups**"

**Lines 288-291**: The highlighted phrase; “**the exposure and protection of malaria**” is confusing and should be clarified.

**Lines 324-327**: The sentence is incoherent and should be recast/rephrased for clarity.

**Tables**: Some revisions to the Tables have been indicated (Please see track changes in reviewed manuscript).

**References**: Okay. However, *Plasmodium falciparum* should be italicized throughout as highlighted in the reviewed manuscript.

Thank you.
